# Modelling climate change impacts on the spatial distribution of anthrax in Zimbabwe

**DOI:** 10.1186/s12889-024-17856-9

**Published:** 2024-02-28

**Authors:** Learnmore John, Munyaradzi Davis Shekede, Isaiah Gwitira, Aldridge Nyasha Mazhindu, Davies Mubika Pfukenyi, Silvester Chikerema

**Affiliations:** 1 Department of Geography Geospatial Sciences and Earth Observation, Faculty of Science, University of Zimbabwe, Harare, Zimbabwe; 2 Department of Geospatial Sciences and Earth Observation, National Geospatial and Space Agency, Number 630 Churchill Road, Mt Pleasant, Harare, Zimbabwe; 3 Department of Veterinary Sciences, Faculty of Animal and Veterinary Sciences, Botswana University of Agriculture and Natural Resources (BUAN), Gaborone, Botswana; 4 Department of Clinical Veterinary Studies, Faculty of Veterinary Science, University of Zimbabwe, Harare, Zimbabwe

**Keywords:** Anthrax, Bioclimatic variables, Habitat suitability, Modelling, Zimbabwe

## Abstract

**Background:**

In Zimbabwe, anthrax is endemic with outbreaks being reported almost annually in livestock, wildlife, and humans over the past 40 years. Accurate modelling of its spatial distribution is key in formulating effective control strategies. In this study, an Ensemble Species Distribution Model was used to model the current and future distribution of anthrax occurrence in Zimbabwe.

**Methods:**

Bioclimatic variables derived from the Beijing Climate Centre Climate System Model were used to model the disease. Collinearity testing was conducted on the 19 bioclimatic variables and elevation to remove redundancy. Variables that had no collinearity were used for anthrax habitat suitability modelling. Two future climate change scenarios for different Representative Concentration Pathways (RCP), RCP4.5 and RCP8.5 were used. Model evaluation was done using true skill, Kappa statistics and receiver operating characteristics.

**Results:**

The results showed that under current bioclimatic conditions, eastern and western districts of Zimbabwe were modelled as highly suitable, central districts moderately suitable and southern parts marginally suitable for anthrax occurrence. Future predictions demonstrated that the suitable (8%) and highly suitable (7%) areas for anthrax occurrence would increase under RCP4.5 scenario. In contrast, a respective decrease (11%) and marginal increase (0.6%) of suitable and highly suitable areas for anthrax occurrence were predicted under the RCP8.5 scenario. The percentage contribution of the predictors varied for the different scenarios; Bio6 and Bio18 for the current scenario, Bio2, Bio4 and Bio9 for the RCP4.5 and Bio3 and Bio15 for the RCP8.5 scenarios.

**Conclusions:**

The study revealed that areas currently suitable for anthrax should be targeted for surveillance and prevention. The predicted future anthrax distribution can be used to guide and prioritise surveillance and control activities and optimise allocation of limited resources. In the marginally to moderately suitable areas, effective disease surveillance systems and awareness need to be put in place for early detection of outbreaks. Targeted vaccinations and other control measures including collaborative ‘One Health’ strategies need to be implemented in the predicted highly suitable areas. In the southern part where a high decrease in suitability was predicted, continued monitoring would be necessary to detect incursions early.

## Background

Anthrax is a zoonotic disease of concern that occurs naturally in herbivorous wildlife and livestock thereby significantly affecting human livelihoods and biodiversity. The disease is one of the neglected tropical diseases which is caused by the gram-positive spore-forming bacterium *Bacillus anthracis* [1]*.* In terms of geographic distribution and endemism*,* anthrax is found in several regions across the globe such as Asia, Australia, North and South America, Southern parts of Europe, sub-Saharan Africa and Central and South America [2, 3]. The spatial distribution of the disease is attributed to the ability of *B. anthracis* to form spores that thrive well under diverse environmental conditions [4]. Although the disease burden of anthrax in herbivores is not fully known, studies have shown that anthrax outbreaks occur almost every year killing thousands of animals and transmitting the disease to humans upon consumption of the meat [1]. The disease is of global concern as it results in high animal mortality with subsequent threats to human health [5, 6]. Despite a decrease in reported livestock anthrax cases globally in the past decade [7] between 20,000 to 100,000 cases of the disease are still being recorded each year especially in developing countries [4]. The disease also affects human beings with 1.83 billion people living within high anthrax-risk areas and Africa recording the highest human incidences of the disease [4]. In fact, human anthrax cases often associated with animal anthrax epidemics in resource poor communities occur at least every year in African countries such as Zambia, Zimbabwe, and Ethiopia [8, 9]. Thus, there is need to develop or adopt methods that allow for better understanding of current and future spatial distribution of anthrax as a preamble to identifying potential anthrax hotspots [10].

Zimbabwe has an estimated cattle herd of ~ 5.5 million with 90% of the national cattle herd under the smallholder sector [11]. Over the years, the national herd has declined due to increased mortality from anthrax and tick-borne diseases such as January disease and Heart water [12]. Previous studies have reported the following cattle deaths emanating from tick-borne diseases in Zimbabwe: 3,430 in 2017; 1,133 in 2018; 1,903 in 2019; 2,772 in 2020 and 1,478 died in 2021 [13]. In fact, it has been reported that cattle deaths from tick-borne diseases can be as high as 9% of the national heard [14]. This is despite an increase in the surveillance and disease control measures to curtail the occurrence and spread of tick-borne diseases by the Department of Veterinary Services [14]. Typical anthrax outbreaks in the country are usually recorded during the dry (July to October) and wet (November to February) seasons.

Anthrax is transmitted via several modes in livestock and wildlife. Ingestion of spores during grazing in landscapes that previously experienced anthrax outbreaks is the primary mode of transmission in animals [15, 16]. Scavenging animals, biting flies or poor disposal of infected animal carcasses facilitate disease transmission through exposing vegetative cells to oxygen thereby resulting in spore formation [17]. Vaccination and proper carcass disposal are the main methods of control in the event of outbreaks.

The spatial distribution of anthrax is influenced by several factors which include livestock density, soil pH, availability of surface water, rainfall, temperature dynamics and vegetation cover [18]. High livestock density increases interaction among individual animals thereby increasing anthrax transmission [19]. The interaction usually occurs as livestock forage for resources including when searching and drinking surface water. The interaction is especially intense during the dry season when there are limited waterholes thereby resulting in increased interaction as livestock from different geographical regions mix unlike in the wet season when water sources are ubiquitous [20]. During the dry season when pastures are scarce and the grass has become shorter, there is a high probability for animals to consume the grass together with soil particles often leading to abrasions in the mouth thus increasing chances of disease transmission in contaminated areas. On the other hand, soils which are slightly alkaline (pH of 6.74) and contains high calcium levels help to maintain the *B. anthracis* spore cell wall integrity. This results in continued persistence of anthrax in endemic areas. Of late, climate change seems to be a key driver influencing anthrax occurrence and distribution [21]. Heavy rains and floods following a long dry period combined with high temperature results in transportation and deposition of *B. anthracis* spores in low-lying areas as well as speeding up the bacterium life cycle [22].

Numerous studies covering different aspects of anthrax have been carried out at different spatial and temporal scales in Zimbabwe. These studies include those that assessed the ecological niche of *B. anthracis* [23, 24] and those that focused on spatial and temporal distribution of anthrax [9, 25], anthrax in animals [26–28] and humans [29–39]. Studies have also assessed influence of politics on anthrax control [40] as well as its impact on rural livelihoods [9]. Although these studies have improved the understanding of anthrax ecology, spread and dynamics in both space and time, they lack futuristic insights into the potential effects of climate change on anthrax occurrence in Zimbabwe. Information on the distribution of anthrax is important for anthrax control and management strategies, such as the targeted vaccinations, optimizing resource allocation and prioritisation of prevention and control strategies in high-risk areas [41]. This is particularly important in a resource-poor country such as Zimbabwe, where the anthrax vaccines are often inadequate to cover all livestock across the country. Therefore, the objectives of this study were to determine the current distribution of anthrax outbreaks as well as predict the future habitat suitability and distribution of anthrax occurrence using bioclimatic predictors. This is important to inform surveillance, control and prevention strategies which need to be undertaken by veterinary and public health personnel.

## Methods

### Study area

**Fig. 1:**
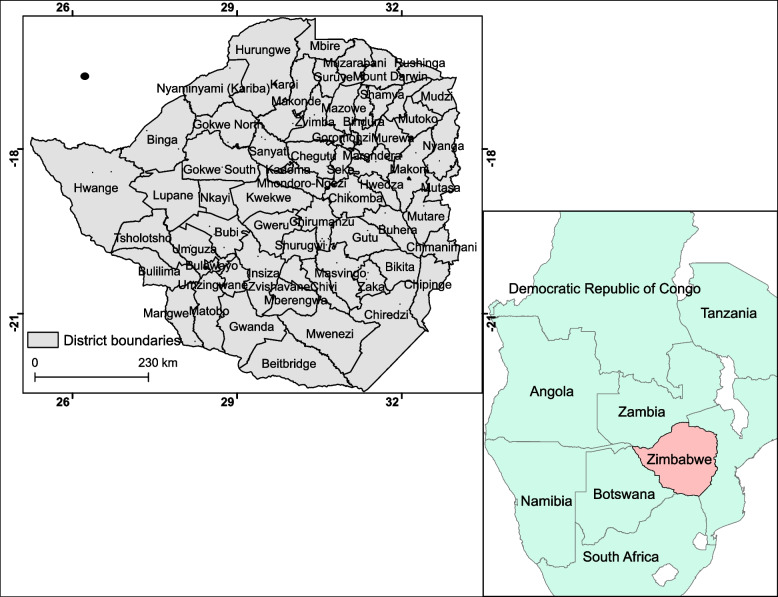
Location of Zimbabwe within the Southern African development community in Africa

The study was conducted in Zimbabwe, a country in southern Africa bound by longitudes 25^0^ E and 34^0^ E and latitudes15^0^ S and 23^0^ S (Fig. 1). Elevation is highest in the eastern parts of the country (> 2500 m above sea level – a.s.l) and lowest in the southern and northern parts of the country where it reaches less than 300 m a.s.l. The climate in the country is characterised by warm-wet months from November to May, cool-dry months (June to August) and hot-dry months (September to November). Annual rainfall is highest (> 1500 mm) in the eastern highlands and lowest in the western and southern parts of the country where it is less than 400 mm. Temperature ranges from an average low of 15 °C in July to around 24 °C in November. The soils are predominantly of granitic origin covering 46% of the country. Zimbabwe is characterized by both extensive and intensive livestock production combined with dryland and irrigated crop farming and vast wildlife conservation areas.

### Data sources

Under the Animal Health and Public Health Acts of Zimbabwe, anthrax is a notifiable disease and reporting all observed and confirmed outbreaks in animals and humans is therefore mandatory [9, 24]. Hence, the surveillance system for anthrax in both the animal and human sectors is based on notification [9]. Furthermore, all district veterinary offices and animal health centres are required to submit animal disease reports to the provincial veterinary offices where the data is collated.

The collated disease information from all the provinces is then submitted to the Information Management Unit (IMU) in the Department of Veterinary Services (DVS) where it is electronically stored. The confirmation is based on clinical signs and microscopic examination of blood smears with no culture [24]. In this study, a total of one hundred and twelve (112) georeferenced data of confirmed cattle anthrax outbreaks from October 2011 up to January 2015 were obtained from the Information Management Unit (IMU) of the DVS. The IMU uses the Military Grid Reference System to georeference the location of anthrax outbreak sites and the data is converted to latitude/longitude using an Excel spreadsheet converter freely available online [24]. The geolocation of some anthrax outbreak sites was also captured using the Global Positioning System (GPS) device. Anthrax outbreaks were identified and defined by their spatio-temporal distance; that is separated by their locality and/or time [9]. This was also cross-checked based on the expertise of veterinary staff who directly followed outbreaks of the disease. Table 1 provides the attributes of the data contained in the anthrax dataset that was used in the study.

**Table 1:** Description of the attributes of the dataset used in modelling potential climate change impacts on the spatial distribution of anthrax

Attribute	Description
Year	The year the disease was reported
Month	The month during which the case/death was reported
Owner’s name	The name of the person owning the livestock
Province	The administrative province in which the case was recorded
District	The administrative district in which the case was recorded
Type of observation	Whether the observation is initial or follow-up to a previously reported case(s)
Type of locality	The environment in which the case was observed ie.e, dip tank, village, grazing area etc
Dip tank	The name of the dip tank from which the case was reported from
Grid Reference	Location on a map expressed in terms of northings and eastings
Tentative diagnosis	A preliminary diagnosis of an animal disease made by a veterinary surgeon physicians according to physical examination and clinical findings
Lab diagnosis	A diagnosis that is based on laboratory reports or test results
Final Diagnostics	Done based on provisional diagnosis and investigations
Infection source	The origin from which a host acquires the infection
Date of onset	The date when the outbreak started
Census	Total population of animals in the district
Cases	Number of reported cases of the disease
Deaths	Number of reported deaths of animals from the disease
Post-mortem	A surgical procedure involving a thorough examination of a carcass by dissection to determine the cause, mode, and manner of death

Apart from the attributes presented in Table 1, the dataset also contained information on the date the anthrax case was reported to the veterinary officer, date for final diagnosis, date interventions started, vaccines used, number of animals treated, sex and age of animal as well as the outbreak status. However, since the interest was on modelling the spatial distribution of anthrax in the context of climate change, only locational data was important. Data on cases and deaths were later used for validating the modelled spatial distribution of anthrax.

### Predictor variables

Twenty predictor variables, which included 19 bioclimatic variables and elevation, were considered for niche modelling (Table 2). The bioclimatic variables for the current climate consisting of temperature and precipitation related factors and their derivatives were freely downloaded from the world climate data website at (http://worldclim.org/version2.1). The bioclimatic variables were based on the Beijing Climate Centre Climate System Model for the current and future climate scenarios i.e., 4.5 and 8.5 Representative Concentration Pathways (RCP) [42]. The term “representative” means that each RCP provides only one of many possible scenarios that result in a particular radiative forcing. RCP4.5 is the intermediate stable path where the radiative forcing stabilizes at approximately 4.5Wm^2^ and 6.0Wm^2^ after 2100. On the other hand, RCP8.5 assumes that the radiative forcing will reach more than 8.5Wm^2^ by 2100 and will continue to increase over a period of time [43]. The data contain 19 variables, consisting of 11 temperature covariates (Bio1-Bio11) and 8 precipitation covariates (Bio 12-Bio 19) (Table 2). The data used are average values for the period 1970–2000 available at a spatial resolution of 30 s i.e., ~ 1 km^2^. The Digital Elevation Model was also downloaded from world climate data website [44].

**Table 2:** Bioclimatic variables used for modelling

Bioclimatic variable	Description
Bio1	Annual mean temperature
Bio2	Mean diurnal range
Bio3	Isothermality
Bio4	Temperature seasonality
Bio5	Maximum temperature of the warmest month
Bio6	Minimum temperature of the coldest month
Bio7	Temperature annual range
Bio8	Mean temperature of wettest quarter
Bio9	Mean temperature of driest quarter
Bio10	Mean temperature of warmest quarter
Bio11	Mean temperature of coldest quarter
Bio12	Annual precipitation
Bio13	Precipitation of wettest month
Bio14	Precipitation of driest month
Bio15	Precipitation seasonality
Bio16	Precipitation of wettest quarter
Bio17	Precipitation of driest quarter
Bio18	Precipitation of warmest quarter
Bio19	Precipitation of coldest quarter
DEM	Digital Elevation Model

### Testing for collinearity

Before modelling the distribution of anthrax outbreaks, the twenty variables were first tested for collinearity using the Variance Inflation Factor (VIF) [44]. Collinearity between environmental variables leads to model overfit [45]. VIF thus provides an estimate of how much variability of a predictor variable is explained by the rest of the predictor variables in the model [46]. On the other hand, correlation analysis provides the strength of relationship between two or more continuous variables. When the predictor variables are correlated, they explain part of the same variance in the dependent variable, thus reducing its statistical significance [47]. As a rule of thumb, VIF values greater than 10 represents collinearity [48]. The spatial distribution of anthrax outbreaks was then modelled under current and future climate after eliminating 11 of the 20 input variables that were highly correlated (Table 3).

**Table 3:** Environmental variables used for modelling the spatial distribution of anthrax after removing highly correlated variables

Variable	VIF
Bio2	7.41
Bio3	3.38
Bio4	9.36
Bio6	6.71
Bio9	9.88
Bio15	4.77
Bio18	2.79
Bio19	3.45
DEM	2.75

### Modelling niche of anthrax

In this study, an ensemble of eight machine learning algorithms was used to model the potential impact of climate change on the distribution of anthrax as well as identify areas at risk in Zimbabwe. Specifically, General Linear Model (GLM), Multiple Adaptive Regression Spline (MARS), Surface Range Envelope (SRE), Generalised Boosted model (GBM), Random Forest (RF), Classification Tree Analysis (CTA), Flexible Discriminant Analysis (FDA) and Maximum Entropy (MaxEnt) were used to predict ecologically suitable anthrax habitats. These SDMs were selected based on their relatively higher predictive power (ROC > 0.6) than the discarded models. To cater for the variability among algorithms, an ensemble modelling approach was used to integrate various SDMs constructed through different modelling algorithms [49]. Ensemble modelling reduces over fitting since it incorporates all different Species Distribution Models (SDMs) to develop an output model [50]. The analysis was performed in R Version 4.1.0 environment (R Core Team, 2019) using the BIOMOD2 package [51]. The model was calibrated using 80% of the occurrence points (presence and pseudo-absence) as training data and 20% of test data for evaluation [52]. One hundred and twelve points of presence data from October 2011 up to January 2015, were used in modelling. To determine overall suitability, the probability of each model output was multiplied by a given weight for different models. After multiplying by weight, each output was divided by the number of models used to get the final probability.

### Model validation

The area under the receiver operating characteristic (ROC) curve, true skill statistic (TSS) and Kappa statistic were used to determine the accuracy of the ensemble model [53]. In this study, the models with greater than fair predictive accuracy i.e., ROC > 0.6 were used to build an ensemble from the individual model outputs. A model with a predictive accuracy of greater than 0.5 is regarded as useful while a ROC of 0.6 is regarded as a better model [54]. Based on the performance of the Ensemble Species Distribution Model, the ensemble model was regarded as suitable in predicting environmental suitability for the anthrax outbreaks in Zimbabwe under different climate scenarios as it had ROC > 0.7, TSS and Kappa > 0.6. The employment of multiple metrics for model validation is a standard practice that ensures the reliability of the model results and helps uncover errors and inconsistencies from different angles [54–58]. For instance, [54] clearly demonstrated how TSS can overcome some of the weaknesses associated with Kappa statistic while preserving the positive aspects of the evaluation technique.

### Change detection

The modelling output maps showing probability of anthrax occurrence were reclassified into nominal classes in ArcGIS Version (10.3.1). Nominal classes were assigned after ranking the probability of occurrence of anthrax, 0–0.25 marginally suitable, 0.251–0.5 moderately suitable, 0.51–0.75 suitable and 0.751–100 highly suitable based on previous studies [59]. An overlay analysis based on current and future suitability of anthrax occurrence was performed to determine where and in what direction suitability of anthrax occurred.

## Results

### Current distribution of anthrax occurrence

**Fig. 2:**
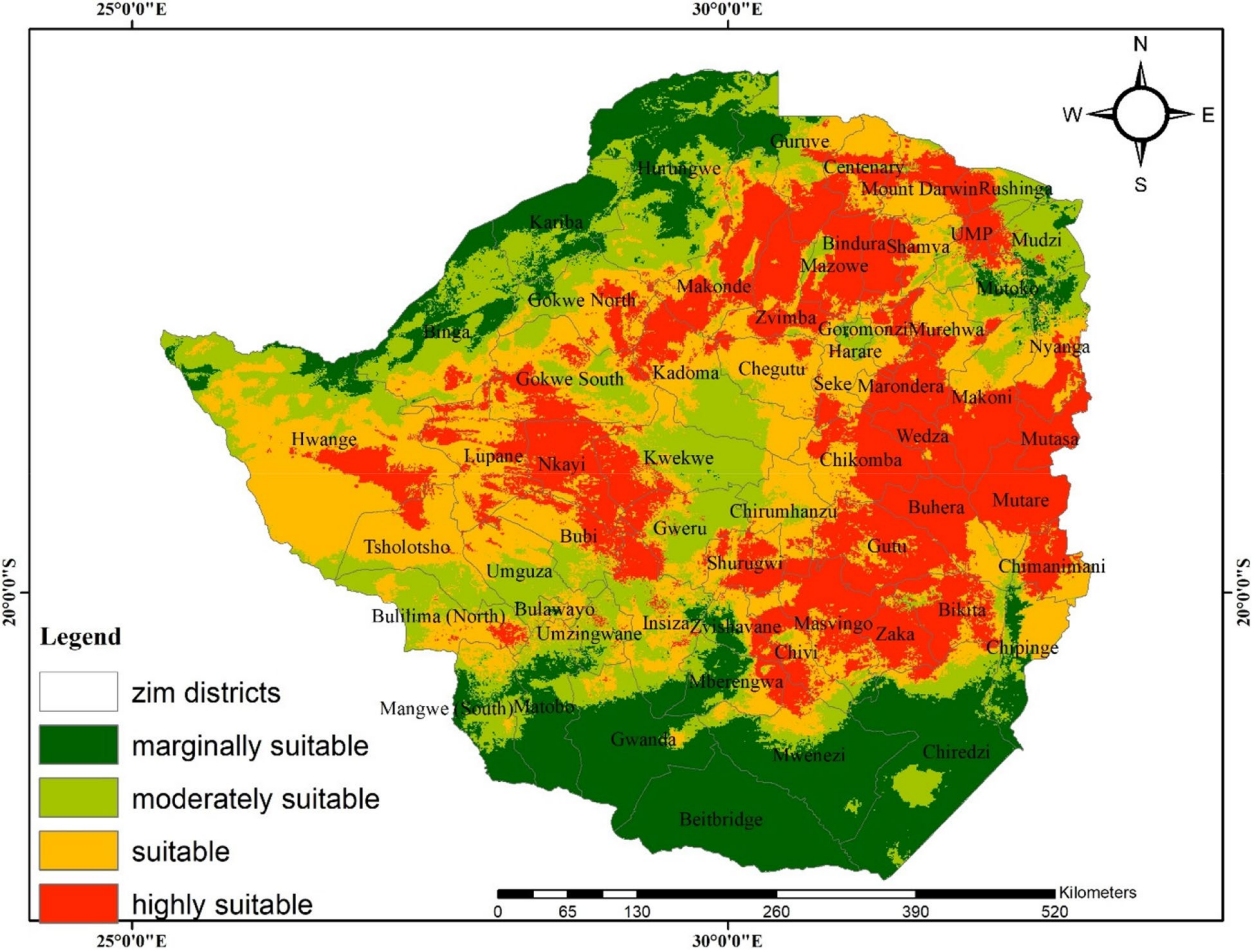
The current modelled distribution of anthrax occurrence in Zimbabwe using bioclimatic variables based on ensemble modelling

The spatial distribution of anthrax modelled under current climate shows that the eastern (Chimanimani, Makoni, Marondera, Mutare, Mutasa and Wedza), northern (Bindura, Makonde, Mazowe and Mt Darwin) and western districts (Hwange, Lupane, Tsholotsho, Bubi and part of Gokwe North) of Zimbabwe are highly suitable for anthrax occurrence (Fig. 2). On the other hand, the central parts of Zimbabwe are moderately suitable for anthrax occurrence with Kadoma, Kwekwe, Gweru, some parts of Chirumhanzu and Gokwe South, Harare, Mudzi and UMP being notable districts (Fig. 2). In contrast, the southern districts (Beitbridge, Gwanda, Mwenezi and Chiredzi) are marginally suitable for the disease occurrence (Fig. 2).

A high correlation is observed between anthrax cases recorded in the country (cattle, livestock and humans) and the modelled current bioclimatically suitable areas for the disease (Table 4).

**Table 4:** Reported human, livestock and wildlife anthrax cases and deaths in Zimbabwe between 2004 and 2023. Please note there are data gaps arising from non-availability of data

District	Human cases	Livestock cases	Wildlife deaths	Year	Source	Modelled Anthrax Suitability
Chiredzi			1500	2004	[9]	Marginal to moderate
Buhera, Chipinge, Mutare, Mutasa	37	2		2011		High
Mbire and Mt Darwin	40	**5**		2011	[60]	High
Hwange			5	2011		Moderate-high
Hurungwe			3	2011		Marginal-moderate
Buhera, Chipinge, Mutare,Mutasa	49	31		2012		High
Makoni	64	180		June 2013- January 2014	[40]	High
Hwange				2015		Moderate-high
Bikita	33			2020	[61]	High
Marondera	10			2020	[61]	High
Gutu	27			2020	[61]	High
Hurungwe	36			2022	[62]	Marginal-moderate

### Future distribution based on the RCP4.5

**Fig. 3:**
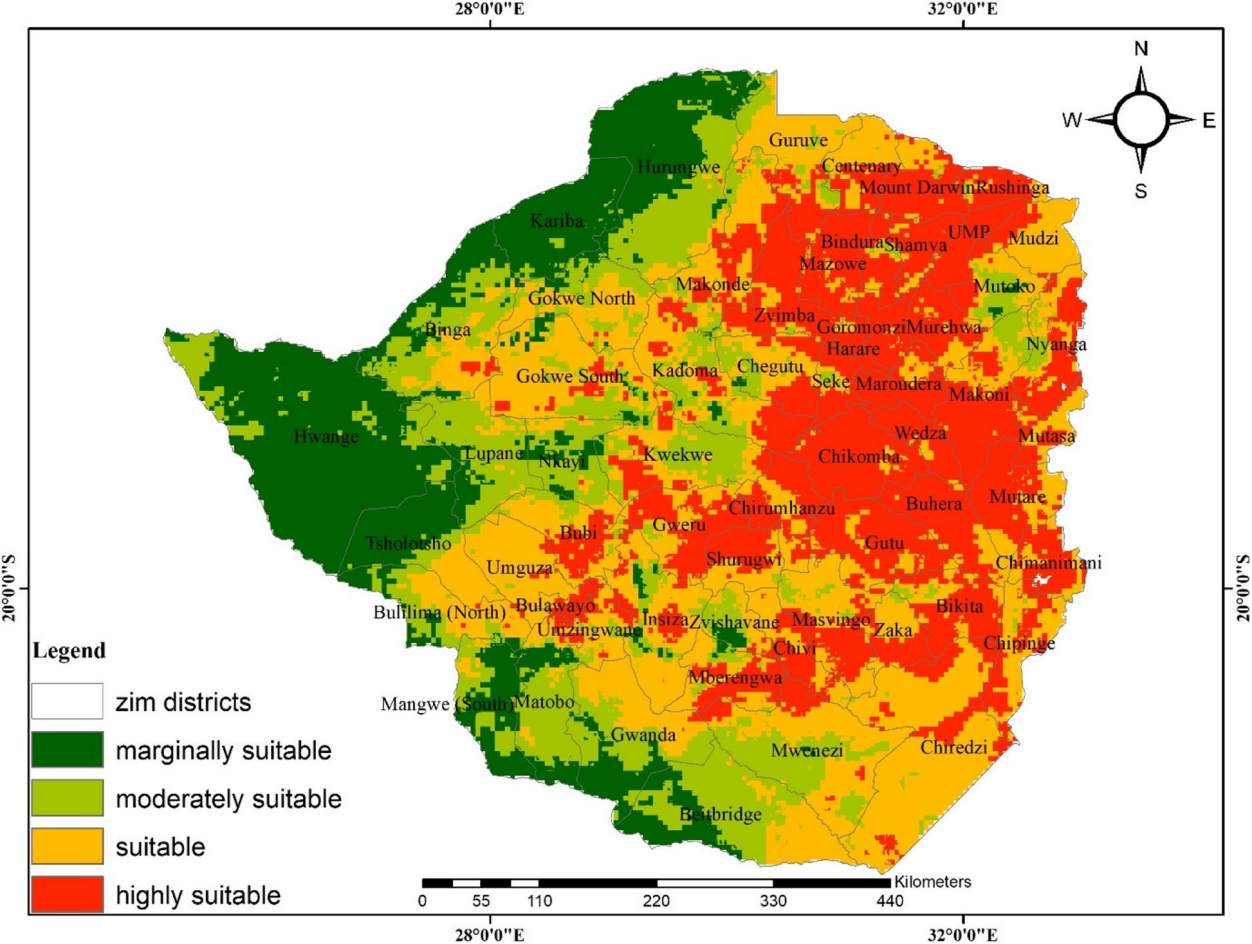
The future projection of anthrax occurrence in Zimbabwe using the Beijing Climate Centre Climate System Model (BCC-CSM-SSP245) under the 4.5 representative concentration pathway

The distribution of anthrax occurrence is projected to significantly expand under the RCP4.5 with the central and eastern parts modelled as highly suitable for the disease. Under this pathway, Nyanga and Chipinge in the eastern parts of the country and UMP, Shamva and Zvimba in the northern parts of the country are expected to increase in both spatial and intensity of suitability (Fig. 3). Conversely, the southern regions which are currently marginally suitable are expected to be suitable for anthrax transmission. In contrast, the western (Hwange and Tsholotsho) and some northern districts are anticipated to become less suitable for the disease.

### Modelled future anthrax distribution under RCP8.5

**Fig. 4:**
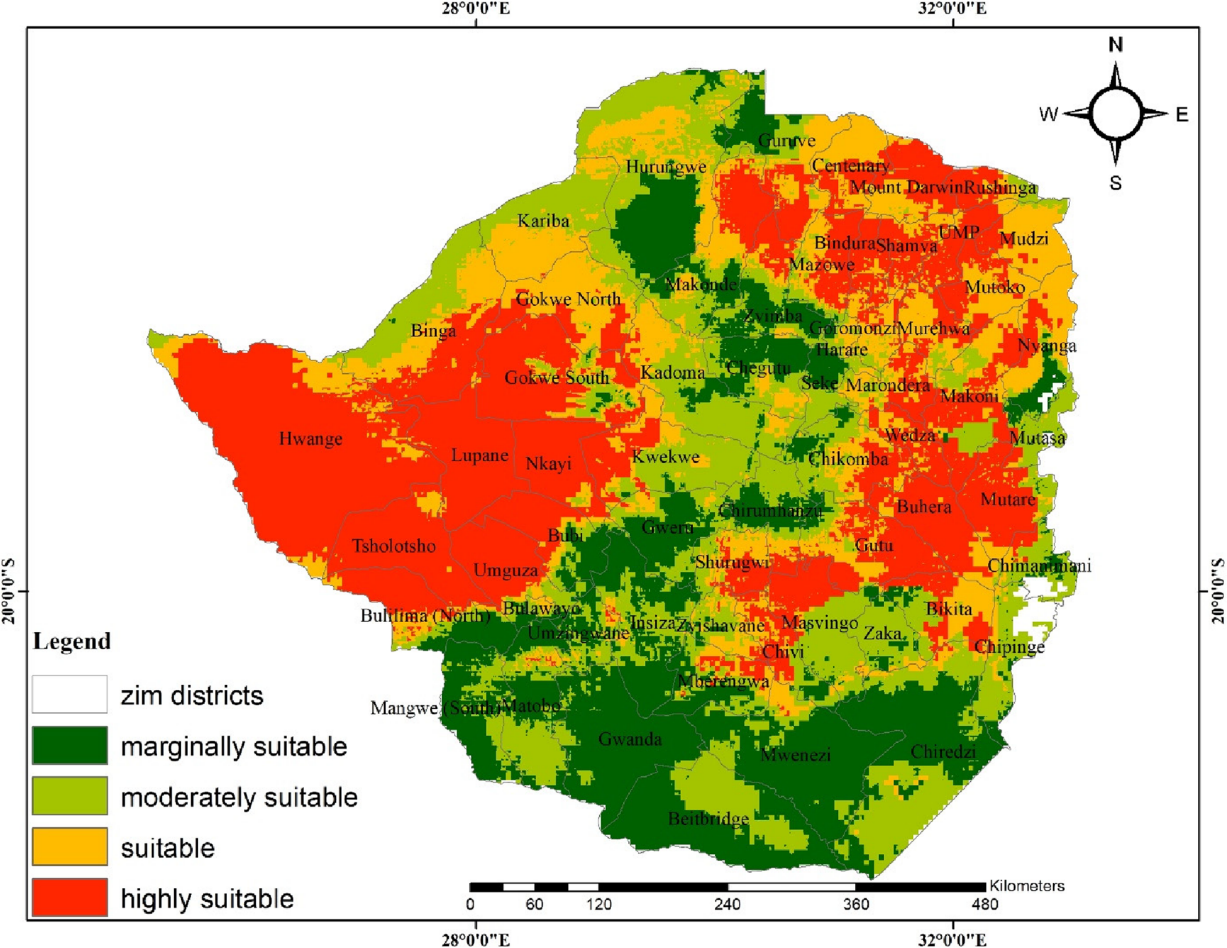
Projected future distribution of anthrax in Zimbabwe using Beijing Climate Centre Climate System Model (BCC-CSM) for the 8.5 Representative Concentration Pathway

Unlike RCP 4.5., anthrax distribution under RCP 8.5 projects a significant shift in suitable regions for athrax occurrence (Fig. 4). Specifically, the western and eastern parts of the country are projected to become highly suitabile for disease transmisssion. The central parts of the country are projected to become less suitable for the disease while the southern parts are generally projected to remain marginally suitable for the disease. Most suitable districts are spatially adjacent to highly suitable areas. Central parts ( Kwekwe and Kadoma) and the eastern parts (Chipinge, Chimanimani and Nyanga) of the country are projected to have moderately suitable conditions for anthrax. Southern (Gwanda, Beitbridge, Chiredzi, Mwenezi, Matobo and Mangwe) and the central parts (Gweru, Chegutu and Zvimba) of the country are projected to be marginally suitable under RCP8.5

**Fig. 5:**
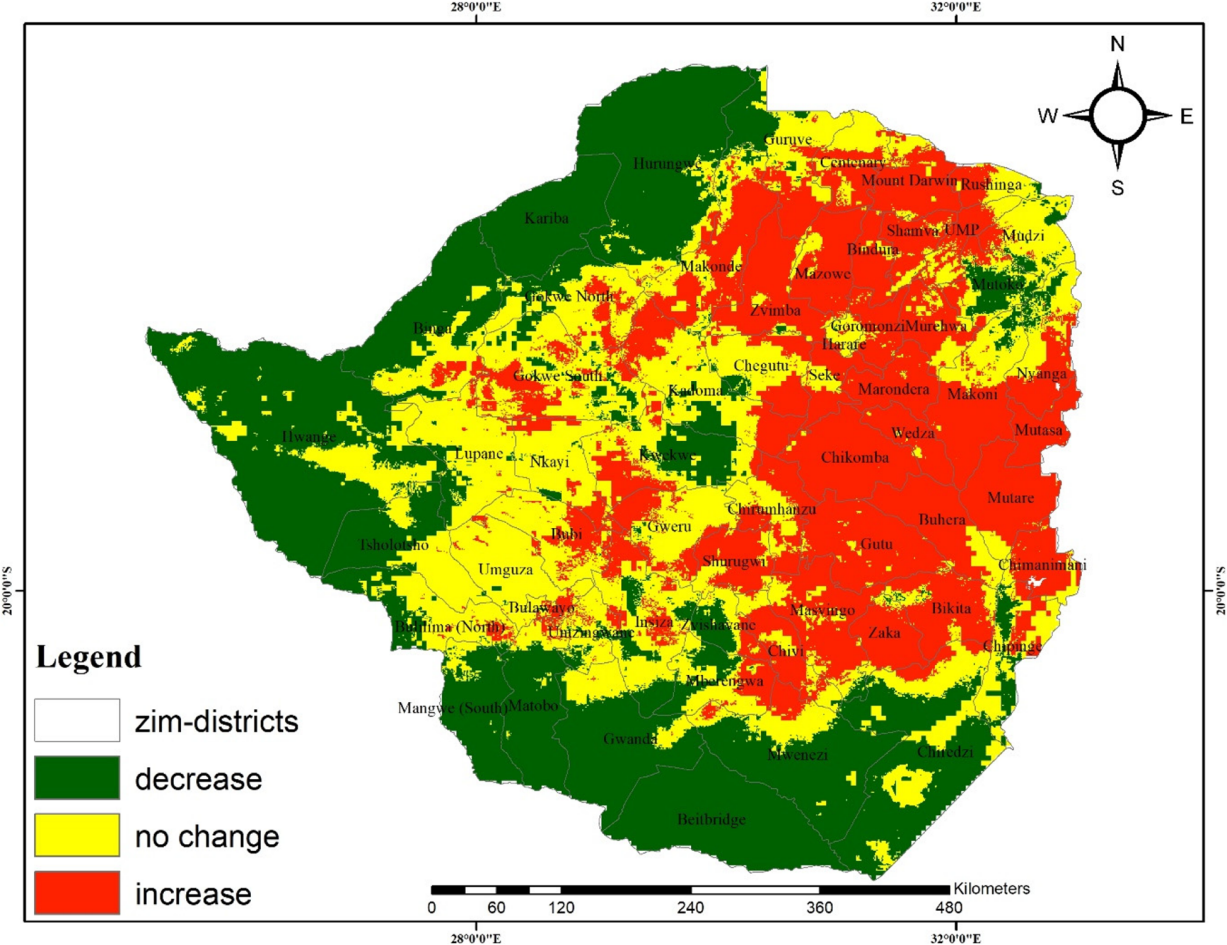
Change detection for environmental suitability for anthrax occurrence under RCP4.5

**Fig. 6:**
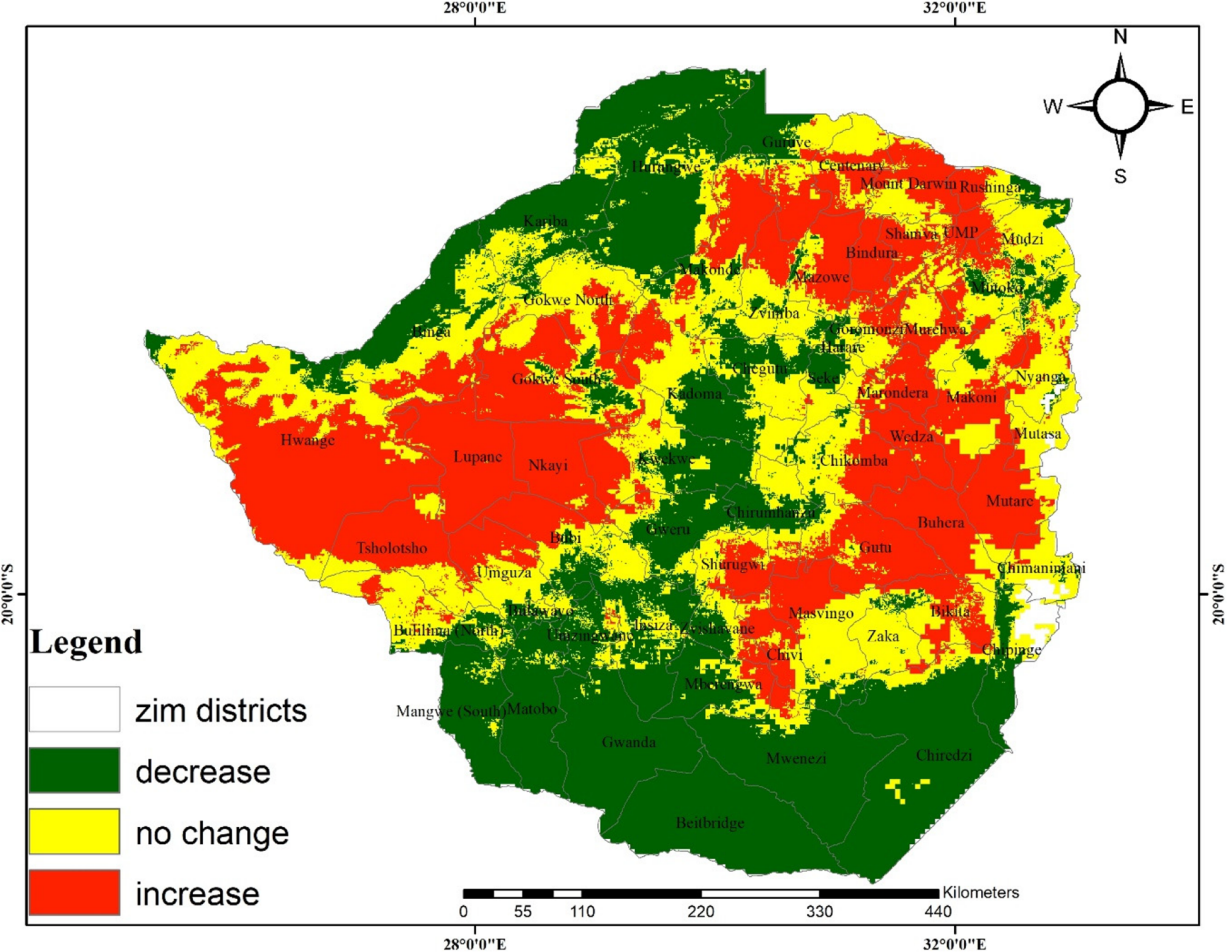
Change detection for environmental suitability for anthrax occurrence under the 8.5 representative concentration pathway

A comparison of current and projected anthrax occurrence under RCPs 4.5 and 8.5 shows contrasting patterns. For instance, suitable and highly suitable areas for the disease are projected to increase by an average of 8% and 7%, respectively (Table 5 and Fig. 5) under RCP 4.5. In contrast, a decrease (11%) and a marginal increase (0.6%) of suitable and highly suitable areas are anticipated under RCP8.5, respectively (Table 5 and Fig. 6). The overall predicted increase in suitable areas for anthrax due to climate change implies likely favorable environmental conditions for the disease leading to its potential range increase which may lead to outbreaks.

**Table 5:** Area and percentage change for suitable and highly suitable projected anthrax distribution

	**Suitable area km^2**	**%Change**	**Highly suitable area km^2**	**%Change**
Current climate	201 199.2		101 308.6	
RCP4.5	232 584.4	8.0	129 314. 7	7.2
RCP8.5	157 746.2	**-**11.1	103 746.1	0.6

Results show a general increase in areas that are suitable for anthrax disease.

### Variable of importance in model

The key predictors of anthrax under the current climate and future RCP (4.5) and RCP (8.5) scenarios were determined using the percentage contribution of each variable to the model (Table 6). Under current climate, Bio18 (precipitation of warmest quarter) and Bio6 (minimum temperature of the coldest month) are the important predictor variables explaining 54.9% of the variation in anthrax occurrence whereas Bio4 (temperature seasonality), Bio9 (mean temperature of driest quarter) and Bio2 (mean diurnal range) become more important under RCP4.5. Bio 3 (isothermality) and Bio15 (precipitation seasonality) are important predictors under RCP8.5. Across the climate scenarios considered in this study, Bio18 and Bio3 have the highest overall gain suggesting that they have the most important information that explains anthrax occurrence if considered on their own. In fact, when Bio18 or Bio3 is not included in the model, the percentage gain is reduced.

**Table 6:** The percentage contribution of the variable for the current and future projections

Variable	Current Percentage (%)	RCP 4.5 Percentage (%)	RCP8.5 Percentage (%)
Bio2	4.8	14.4	5.3
Bio3	3.4	12.4	31.7
Bio4	4.8	21.3	8.3
Bio6	14.6	6.5	3.7
Bio9	5.2	17.6	1
Bio15	10.6	9.4	26.6
Bio18	40.3	4.1	3.4
Bio19	7.5	3.5	9.9
DEM	8.8	10.2	10.2

## Discussion

Results of this study predicted highly suitable areas for anthrax outbreaks in the western and eastern parts of Zimbabwe. The current suitability map shows an increase in highly suitable areas of anthrax compared to previous studies. These results suggest an increase in bioclimatically suitable areas for the disease as well as the superiority of ensemble modelling that integrated eight species distribution models over a single species distribution model (MAXENT), e.g., [25]. In contrast, the study predicted that the northern parts of the country would remain marginally suitable, suggesting that these areas could be less likely to experience anthrax outbreaks and therefore may require less attention relative to other districts. However, the overall results suggest a variable increase in future distribution of anthrax occurrence thereby requiring monitoring of the disease to reduce its impacts [33]. Vaccinations are still one of the best methods to control anthrax and livestock should be vaccinated annually to reduce the incidence of the disease [9]. The first step in implementing vaccination is to determine the priority areas to target hence maps generated in this study can be used to for targeted surveillance and vaccination in the country factoring in their different challenges [63]. Therefore, resources could be channelled towards areas that are projected to be suitable for anthrax in the country [64].

Although this study used cattle anthrax outbreaks only, the models were able to predict wildlife areas such as Hwange National Park as suitable for the disease. However, it is well known that the entire periphery and the interior of a wildlife area is usually shared by livestock and wild animals, and hence the possibility of increased anthrax transmission [65]. This means that the distribution might be expanded if there is spatial overlap between wildlife and livestock which is a common phenomenon at wildlife-livestock interfaces. The areas close to wildlife were predicted to be highly suitable in the future and thus need close monitoring and strategic vaccinations to prevent and reduce the likely future anthrax outbreaks [66]. Previous research in Kenya predicted anthrax occurrence in entire wildlife sanctuaries such as Nakuru National Park [67]. Similarly, the present forecast maps predicted anthrax occurrence in entire Zimbabwean wildlife areas.

From this study, the occurrence and distribution of anthrax was observed to be related to various climate variables. For example, precipitation of warmest quarter (Bio18), minimum temperature of the coldest month (Bio6) and precipitation seasonality (Bio15) were more important in modelling the current distribution of anthrax. Similar findings were observed in western Uganda and Western Africa where seasonality of precipitation and temperature in the warmest months were found to affect the distribution of anthrax [1, 44]. Previous studies by Chikerema et al. [25] showed an increased anthrax outbreak occurrence during the hot dry months in Zimbabwe. This supports findings of this study where precipitation of the warmest quarter contributed more to disease occurrence. Districts such as Beitbridge, Gwanda, Mwenezi, Chiredzi and Kariba were found to be marginally suitable for anthrax occurrence in both the current and future models. This might be due to low precipitation in these districts. Chikerema et al. [25] reported rainfall as a contributing factor for the temporal and spatial occurrence of anthrax in Zimbabwe. The moisture provided by precipitation influences anthrax occurrence through exposing buried spores, collecting and concentrating spores in storage areas, and dispersing spores through runoff. The duration of the dry season is also related to anthrax occurrence. In addition, animals that graze short grasses close to the ground during the dry season are more likely to be exposed to spores thereby increasing the possibility of anthrax outbreaks. The dry season also results in water and forage shortages leading to a higher anthrax transmission in livestock and wildlife at remaining water points [6].

Unlike previous studies, this study used ensemble modelling to assess the potential effects of climate change on the spatial distribution of anthrax. An Ensemble of eight different SDMs was used to understand distribution of anthrax which has an advantage of reducing omission and commission errors since all the prediction of the eight different models were taken into consideration [68]. Ensemble modelling improves model performance resulting in better accuracy compared with a single predictive model [69]. This is achieved through reducing the variance component of the prediction error. An ensemble model can make better predictions and perform better than any contributing model [70]. Another important benefit of the ensemble method is a more robust or reliable average performance of the model. Robustness and reliability are the main concerns in machine learning projects. An ensemble reduces the spread or dispersion of the predictions [71].

This study took into consideration bioclimatic parameters and elevation in modelling anthrax occurrence distribution in Zimbabwe. Future studies should include other environmental factors such as livestock density, soil pH and type, vegetation cover and type and water sources distribution to determine their influence on anthrax occurrence. Factors such as soil pH and type influence the survival of anthrax spores [72] and earlier studies in the country identified soil type as an important predictor followed by variance of vegetation biomass and maximum temperature [24]. Furthermore, the occurrence of anthrax in endemic areas is usually associated with pasture degeneration caused by over utilization or drought. The condition of the pastures leads to nutritional stress and herbivores are forced to feed on heavily utilized short grass or herbs and thereby contract anthrax through ingesting soil containing the spores. Hence, the height of the grass in the grasslands and wooded areas has an influence on the occurrence of anthrax outbreaks. On the other hand, burning is regarded as one of the preferred method of anthrax control through their extermination of most spores as was demonstrated during the massive wildlife anthrax outbreak in Zimbabwe [27]. The method was used to disinfect the soil and vegetation and thereby avoid animals using areas of potentially high contamination and the same approach has been used in the Kruger National Park to sanitize the environment leading to a rapid decrease in the number of deaths due to anthrax outbreaks. Hence, it is likely that annual and extensive bush or grassfires might have an influence on the occurrence of anthrax. These environmental variables are important in the transmission dynamics of the disease and their inclusion in future studies may provide a more accurate potential predicted distribution. Although information on anthrax cases in human, livestock and wildlife is critical for achieving ONE Health, the information is not readily available in a consolidated format and would be critical for informing anthrax management policies and interventions.

## Conclusion

This study predicted current and future occurrence of anthrax outbreaks by geographic area and species under given environmental and climatic parameters. The results projected a respective increase and decrease in suitable areas under the RCP4.5 and RCP8.5 scenarios. However, an overall increase in highly suitable areas was predicted under the two climatic scenarios. The results of this study showed current and future suitable areas of the disease that should be targeted for surveillance, control and prevention. The predicted current and future anthrax distribution can be used as a tool to tackle anthrax. Different interventions or strategies can be developed and applied across the country to minimize predicted future (2040) climate change impacts. In the marginally to moderately suitable areas, effective disease surveillance systems and awareness campaigns need to be put in place for early detection of anthrax outbreaks. Vaccination and control measures including collaborative One Health strategies need to be implemented in those areas predicted to be highly suitable. In the southern part of the country with a predicted high decrease in suitability of anthrax occurrence, continued monitoring would be necessary to detect incursions early. The predictive models and associated results provide valuable information that can be used to develop new spatially explicit prevention and control strategies for anthrax outbreaks in the context of climate change.

## Data Availability

Data are available upon reasonable request from the corresponding author.

## Abbreviations

Bio: Bioclimatic
BCCCSM: Beijing Climate Centre Climate System Model
CTA: Classification Tree Analysis
DEM: Digital Elevation Model
FDA: Flexible Discriminant Analysis
GBM: Generalised Boosted model
GLM: General Linear Model
IMU: Information Management Unit
SDMs: Species Distribution Models
GPS: Global Positioning System
ROC: Receiver operating characteristic curve
TSS: True skill statistic
VIF: Variance Inflation Factor
MARS: Multiple Adaptive Regression Spline
SRE: Surface Range Envelope
RF: Random Forest
MaxEnt: Maximum Entropy
RCP: Representative Concentration Pathways
